# Estimated cardiorespiratory fitness and incident risk of cardiovascular disease in China

**DOI:** 10.1186/s12889-023-16864-5

**Published:** 2023-11-24

**Authors:** Yuanjiao Liu, Jinghan Zhu, Ziye Guo, Jiazhou Yu, Xuhui Zhang, Huiqing Ge, Yimin Zhu

**Affiliations:** 1 Department of Epidemiology & Biostatistics, Zhejiang University School of Medicine, Hangzhou, Zhejiang China; 2 The Second School of Clinical Medicine, Southern Medical University, Guangzhou, Guangdong China; 3 Jockey Club School of Public Health and Primary Care, Chinese University of Hong Kong, Hong Kong, Hong Kong Special Administrative Region China; 4 Hangzhou Center for Disease Control and Prevention, Hangzhou, China; 5 Department of Respiratory Care of Sir Run Shaw Hospital, Zhejiang University School of Medicine, Hangzhou, China; 6 Cancer Center, Zhejiang University, Zhejiang, China

**Keywords:** Cardiorespiratory fitness, Non-exercise testing, Heart disease, Stroke, Prospective cohort study

## Abstract

**Background:**

Limited evidence is available on the association between estimated cardiorespiratory fitness (e-CRF) and incidence of cardiovascular disease (CVD) in Chinese population.

**Methods:**

A total of 10,507 adults including 5084 men (48.4%) and 5423 (51.6%) women with a median age of 56.0 (25% quantile: 49, 75% quantile 63) years from the China Health and Retirement Longitudinal Study (CHARLS) was recruited in 2011 as baseline. The CVD incident events were followed-up until 2018. e-CRF was calculated from sex-specific longitudinal non-exercise equations and further grouped into quartiles. Cox proportional models were used to calculate hazard ratio (HR) and 95% confidence interval (CI) for incidence risks of CVD, heart disease and stroke.

**Results:**

During a median follow-up of 7 years, a total of 1862 CVD, 1409 heart disease and 612 stroke events occurred. In fully adjusted models, each one MET increment of e-CRF was associated with lower risk of CVD (HR = 0.91, 95%CI = 0.85–0.96 for males, HR = 0.87, 95%CI = 0.81–0.94 for females). Compared with the Quartile (Q)1 group, the HRs (95%CI) of the Q2, Q3 and Q4 groups were 0.84 (0.63–1.03), 0.72 (0.57–0.91) and 0.66 (0.51–0.87) for CVD in males. Females had HRs of 0.79 (0.66–0.96) in Q2, 0.71 (0.57–0.88) in Q3 and 0.58 (0.45–0.75) in Q4 for CVD. The associations between e-CRF and heart disease and stroke were slightly weaker than that for CVD in both males and females.

**Conclusions:**

Higher e-CRF decreases the incident risk of CVD, heart disease and stroke.

## Introduction

Cardiorespiratory fitness (CRF) is an integrated indicator that reflects the overall ability to acquire, transport and utilize oxygen during physical activity, which is quantified by maximal oxygen uptake [1]. Higher CRF is associated with lower risk of incidence of cardiovascular disease (CVD) and mortality from CVD [2, 3]. In a meta-analysis of 34 cohort studies, per 1 metabolic equivalent (MET) increase in CRF was associated with 13% (9–17%) lower risk of CVD mortality [4]. Cardiopulmonary exercise testing with ventilatory gas analysis at maximal effort is considered to be the gold standard of assessing CRF [5]. Alternatively, CRF can be measured by submaximal exercise tests using a specific physiological response with a standardized protocol (e.g., heart rate [HR], peak workload, amount of time or distance covered running or walking) [6]. These methods could provide relative accurate measurement of CRF, however, the use of them is limited as the high cost of specialized equipment, trained staff, a large indoor space, and a lot of time. Only individuals with relative healthy condition and normal exercise performance could complete the CRF test. Several non-exercise CRF estimation algorithms based on easily measured and self-reported variables have been developed to simplify the access to CRF data, especially in large population investigation. The application of CRF could be expanded to participants with limited mobility or chronic diseases.

Most algorithms were derived from cross-sectional studies with linear regression of age. Longitudinal data showed that CRF declines nonlinearly with aging [12, 13]. To estimate CRF more accurately, Jackson et al. developed a group of sex-specific longitudinal non-exercise equations accounting for the age-associated decline in CRF based on common physical parameters [14]. In Aerobics Center Longitudinal Study (ACLS), e-CRF based on longitudinal algorithms provided a valid prediction of all-cause mortality, CVD-related mortality and non-fatal CVD events, which was consistent with the results based on measured CRF [15]. Besides, e-CRF may add clinical value to the Framingham Risk Score to better predict long-term coronary heart disease (CHD) risk in males from ACLS [19]. Higher e-CRF estimated by longitudinal equations was associated with lower risk of all-cause and CVD mortality in both sexes derived from Third National Health and Nutrition Examination Survey [16]. In Norway population, e-CRF was a strong predictor of mortality from CVD an all-cause independent of traditional CVD risk factors [18]. Another study based on the same Norway population illustrated that e-CRF lower the risk of first acute myocardial infarction among females but not males [20]. Higher non-exercise CRF was related to lower death risk in women but not in men of Spanish older population [6]. The evidence on the association between e-CRF and inverse outcomes in Chinese population was limited. In Taiwan MJ Cohort, both e-CRF and its changes predicted the incidence of major biological CVD risk factors, especially hypertension and type 2 diabetes mellitus (T2DM) [21]. e-CRF was inversely associated with risk of all-cause and cause-specific mortality in The Rural Chinese Cohort Study [17]. However, no reports are available for the association between e-CRF and incidence of CVD including heart disease and stroke in Chinese population.

Thus, the aim of this study was to examine the associations between e-CRF and incidence of CVD, heart disease and stroke in a large prospective cohort in China. This study would provide evidence for application of e-CRF in different populations and inform future public health recommendations.

## Methods

### Study population

The data used in this study were downloaded from the China Health and Retirement Longitudinal Study (CHARLS, available at CHARLS (pku.edu.cn)). CHARLS is an ongoing national and household-based cohort survey of Chinese adults aged 45 years above. The baseline investigation started in 2011 and being followed up every 1–2 years. By now, CHARLS has been conducted for five Waves (in 2011, 2013, 2014, 2015 and 2018 respectively), among which the Wave1 (2011) and Wave4 (2015) collected blood samples and the Wave3 (2014) is the Life History Survey. Briefly, CHARLS was conducted using a multistage probability sampling and covered 28 provinces across Mainland China. Details about the research design and implementation process of CHARLS are available elsewhere [22]. The face-to-face interviews, anthropometric measurements and blood samples tests were conducted by trained staff following standard protocols. In this study, we used data in 2011 as baseline and followed up until 2018. [22]The primary aim of this study was to recruit subjects aged ≥ 45 years, but some subjects aged 18–44 years also attended the baseline survey. As several CRF estimation components were derived from anthropometric measurements, we included the individuals participated in biomarker measurement at least once in 2011, 2013 and 2015 to maximum the sample size. A nationally representative sample of the 15,292 subjects was recruited in Wave1. Subjects who met the following criteria were excluded: (1) missing data on e-CRF components (age, sex, BMI, WC, rHR, physical activity level, smoking), (2) had heart disease and stroke at baseline, (3) loss to follow-up. Ultimately, a total of 10,507 participants were include into this study. Ethics approval for the data collection was obtained from the Biomedical Ethics Review Committee of Peking University.

### Background characteristics and anthropometric measurements

Participants were interviewed face-to-face using a structured questionnaire, which asked for demographic information (e.g., date of birth, sex, education attainment,), lifestyle data (e.g., smoking, alcohol use, physical activity), self-reported diseases (heart disease, stroke, hypertension, dyslipidemia, diabetes, and cancer) and use of medications for corresponding diseases. In this study, smoking/ drinking were defined as current or previous smoking/drinking. Each subject reported their amount of physical activity in a usual week including vigorous physical activity (VPA), moderate physical activity (MPA) and light physical activity (LPA). VPA makes participants breathe much harder than normal and may include heavy lifting, digging, plowing, aerobics, fast bicycling, and cycling with a heavy load. MVPA makes participants breathe somewhat harder than normal and may include carrying light loads, bicycling at a regular pace, or mopping the floor. LPA includes walking at work and at home, walking to travel from place to place, and any other walking that participants might do solely for recreation, sport, exercise, or leisure. Subjects who had conducted VPA/MPA/LPA for at least 10 min continuously in a usual week were further asked about the frequency and duration of VPA/MPA/LPA. Conducting a total of VPA or MPA greater than 4 days per week was considered as active physical activity.

Anthropometric indexes including weight, height, WC, rHR, systolic blood pressure (SBP) and diastolic blood pressure (DBP) were measured by trained staff following a standard protocol. BMI was calculated as weight (kg)/height (m) [2]. Hypertension was diagnosed by the following criteria: (1) SBP ≥ 140 mmHg and/or DBP ≥ 90 mmHg, (2) any treatment for hypertension, and (3) self-reported history of hypertension diagnosed by clinical physicians.

**Table 1:** Baseline characteristics of the participants according to the quartile of e-CRF by men and women

Characteristics	Q1	Q2	Q3	Q4	*P* value	
**Total population (n)**	2,627	2,626	2,627	2,627		
Age, mean (SD), year	65.9 (10.1)	57.4 (8.15)	54.4 (7.15)	51.2 (6.03)	< 0.001	
Female, n (%)	1356 (51.6)	1355 (51.6)	1356 (51.6)	1356 (51.6)	1	
Rural Area, n (%)	1418 (54.0)	1570 (59.8)	1716 (65.3)	1804 (68.7)	< 0.001	
Smoking, n (%)	1171 (44.6)	1078 (41.1)	1022 (38.9)	915 (34.8)	< 0.001	
Drinking, n (%)	1091 (41.5)	1135 (43.2)	1128 (42.9)	1070 (40.7)	0.214	
Active physical activity, n (%)	217 (8.3)	394 (15.0)	587 (22.3)	1029 (39.2)	< 0.001	
BMI, mean (SD), kg/m ^2^	26.1 (4.73)	24.6 (3.25)	23.0 (2.62)	21.1 (2.28)	< 0.001	
WC, mean (SD), cm	92.7 (11.3)	88.0 (10.1)	83.0 (9.40)	76.0 (11.5)	< 0.001	
rHR, mean (SD), bpm	77.4 (11.2)	74.8 (9.94)	71.7 (9.04)	67.8 (8.43)	< 0.001	
Hypertension, n (%)	1587 (60.5)	1132 (43.2)	815 (31.2)	512 (19.6)	< 0.001	
SBP, mean (SD), mmHg	138.8 (21.3)	131.5 (20.0)	126.3 (19.1)	121.5 (17.7)	< 0.001	
DBP, mean (SD), mmHg	78.9 (12.5)	77.8 (12.3)	75.6 (12.0)	72.6 (11.2)	< 0.001	
TG, mean (SD), mmol/L	1.72 (1.13)	1.64 (1.12)	1.41 (0.91)	1.24 (0.78)	< 0.001	
HDL-C, mean (SD), mmol/L	1.24 (0.35)	1.27 (0.36)	1.35 (0.36)	1.42 (0.38)	< 0.001	
FPG, mean (SD), mmol/L	6.32 (1.96)	6.09 (1.74)	5.81 (1.49)	5.60 (1.30)	< 0.001	
Drug for hypertension, n (%)	658 (25.0)	382 (14.5)	246 (9.4)	126 (4.8)	< 0.001	
Drug for dyslipidemia, n (%)	162 (6.2)	107 (4.1)	61 (2.3)	35 (1.3)	< 0.001	
Drug for diabetes, n (%)	133 (5.1)	88 (3.4)	50 (1.9)	29 (1.1)	< 0.001	
e-CRF, METs, mean (SD)	8.33 (1.56)	9.85 (1.42)	10.7 (1.55)	11.7 (1.77)	< 0.001	
**Men (n)**	1,271	1,271	1,271	1,271		
Age, mean (SD), year	64.8 (10.3)	58.0 (8.66)	55.7 (7.34)	52.7 (6.03)	< 0.001	
Rural Area, n (%)	660 (51.9)	754 (59.3)	852 (67.0)	918 (72.2)	< 0.001	
Smoking, n (%)	984 (77.4)	957 (75.3)	936 (73.6)	875 (68.8)	< 0.001	
Drinking, n (%)	872 (68.6)	901 (70.9)	909 (71.5)	863 (67.9)	0.138	
Active physical activity, n (%)	117 (9.2)	200 (15.7)	299 (23.5)	467 (36.7)	< 0.001	
BMI, mean (SD), kg/m ^2^	26.0 (4.31)	24.0 (2.92)	22.5 (2.36)	20.7 (2.04)	< 0.001	
WC, mean (SD), cm	93.6 (10.3)	88.1 (9.63)	82.9 (8.90)	75.2 (12.2)	< 0.001	
rHR, mean (SD), bpm	77.5 (11.8)	75.2 (10.6)	71.7 (9.74)	67.9 (9.10)	< 0.001	
Hypertension, n (%)	727 (57.3)	567 (44.6)	412 (32.6)	278 (21.9)	< 0.001	
SBP, mean (SD), mmHg	137.4 (20.5)	132.5 (19.4)	127.6 (18.2)	123.8 (17.8)	< 0.001	
DBP, mean (SD), mmHg	79.7 (12.9)	78.9 (12.9)	76.5 (12.2)	74.0 (11.5)	< 0.001	
TG, mean (SD), mmol/L	1.72 (1.20)	1.57 (1.12)	1.37 (0.91)	1.19 (0.76)	< 0.001	
HDL-C, mean (SD), mmol/L	1.18 (0.35)	1.26 (0.39)	1.34 (0.38)	1.42 (0.42)	< 0.001	
FPG, mean (SD), mmol/L	6.33 (1.95)	6.09 (1.85)	5.92 (1.64)	5.63 (1.39)	< 0.001	
Drug for hypertension, n (%)	287 (22.6)	167 (13.1)	113 (8.9)	66 (5.2)	< 0.001	
Drug for dyslipidemia, n (%)	81 (6.4)	48 (3.8)	28 (2.2)	17 (1.3)	< 0.001	
Drug for diabetes, n (%)	70 (5.5)	39 (3.1)	30 (2.4)	13 (1.0)	< 0.001	
e-CRF, METs, mean (SD)	9.63 (0.96)	11.3 (0.31)	12.3 (0.28)	13.5 (0.56)	< 0.001	
**Women(n)**	1,356	1,355	1,356	1,356		
Age, mean (SD), year	67.0 (9.80)	56.8 (7.60)	53.2 (6.77)	49.8 (5.70)	< 0.001	
Rural Area, n (%)	758 (55.9)	816 (60.2)	864 (63.7)	886 (65.3)	< 0.001	
Smoking, n (%)	187 (13.8)	121 (8.9)	86 (6.3)	40 (2.9)	< 0.001	
Drinking, n (%)	219 (16.2)	234 (17.3)	219 (16.2)	207 (15.3)	0.569	
Active physical activity, n (%)	100 (7.4)	194 (14.3)	288 (21.2)	562 (41.4)	< 0.001	
BMI, mean (SD), kg/m ^2^	26.2 (5.09)	25.1 (3.44)	23.5 (2.76)	21.5 (2.41)	< 0.001	
WC, mean (SD), cm	91.9 (12.2)	87.9 (10.5)	83.1 (9.85)	76.7 (10.8)	< 0.001	
rHR, mean (SD), bpm	77.3 (10.6)	74.4 (9.24)	71.7 (8.33)	67.7 (7.74)	< 0.001	
Hypertension, n (%)	860 (63.5)	565 (41.8)	403 (29.8)	234 (17.4)	< 0.001	
SBP, mean (SD), mmHg	140.0 (22.0)	130.5 (20.4)	125.1 (19.7)	119.4 (17.4)	< 0.001	
DBP, mean (SD), mmHg	78.1 (12.0)	76.7 (11.6)	74.7 (11.9)	71.3 (10.8)	< 0.001	
TG, mean (SD), mmol/L	1.71 (1.06)	1.70 (1.11)	1.44 (0.91)	1.29 (0.79)	< 0.001	
HDL-C, mean (SD), mmol/L	1.29 (0.34)	1.28 (0.34)	1.36 (0.33)	1.41 (0.35)	< 0.001	
FPG, mean (SD), mmol/L	6.31 (1.97)	6.08 (1.63)	5.72 (1.34)	5.56 (1.20)	< 0.001	
Drug for hypertension, n (%)	371 (27.4)	215 (15.9)	133 (9.8)	60 (4.4)	< 0.001	
Drug for dyslipidemia, n (%)	81 (6.0)	59 (4.4)	33 (2.4)	18 (1.3)	< 0.001	
Drug for diabetes, n (%)	63 (4.6)	49 (3.6)	20 (1.5)	16 (1.2)	< 0.001	
e-CRF, METs, mean (SD)	7.12 (0.89)	8.51 (0.24)	9.23 (0.19)	10.1 (0.40)	< 0.001	

Note: Data are mean (standard deviation) or number (%)

Smoking and drinking: Both current and previous smoking or drinking were defined as smoking or drinking in the multivariable regression

Active physical activity: the threshold of total vigorous physical activity and moderate physical activity greater than 4 days per week was considered as active physical activity

Hypertension: (1) SBP ≥ 140 mmHg and/or DBP ≥ 90 mmHg, (2) any treatment for hypertension, and (3) self-reported history of hypertension, which were previously diagnosed by clinical physicians

The whole blood and serum samples were collected from each subject after a 12-h overnight fasting. Biochemical variables including TG, total cholesterol (TC), HDL-C and low-density lipoprotein cholesterol (LDL-C) and fasting plasma glucose (FPG) were tested in metabolic examinations.

### Assessment of e-CRF

e-CRF in METs was estimated using the sex-specific BMI models constructed by Jackson et al. [14]. These models have been validated in adults and the generated e-CRF have shown association with long-term health risk ^6,15–17,21,23^. The models are as follows:

Male: e-CRF (METs) = 21.2870 + (age × 0.1654) – (age ^2^ × 0.0023) – (BMI × 0.2318) – (WC × 0.0337) – (rHR × 0.0390) + (active physical activity × 0.6351) – (smoker × 0.4263);

Female: e-CRF (METs) = 14.7873 + (age × 0.1159) – (age ^2^ × 0.0017) – (BMI × 0.1534) – (WC × 0.0088) – (rHR × 0.0364) + (active physical activity × 0.5987) – (smoker × 0.2994).

Active physical activity = 1 if the participant is classified as active physical activity or 0 if not. Smoker = 1 if the participant is current or ever smoker or 0 if not.

### Ascertainment of outcome

In this study, CVD includes heart disease and stroke. Heart disease was defined as heart attack, CHD, angina, congestive heart failure or other heart problems. Heart disease and stroke were assessed by asking the participant whether they had been diagnosed with any heart disease/stroke by a doctor and whether they had been under treatment because of heart disease/stroke?” Participants who reported either of the above were classified as incident heart disease/stroke cases. The diagnosis date of incident heart disease or stroke cases was determined by the date between the time of the survey that first recorded heart disease/stroke patients and the time of the previous round of survey.

### Statistical analyses

After applying the algorithms of e-CRF, subjects were divided into four groups according to sex-specific quartile. Continuous variables were described as mean (standard deviation, [SD]) and were analyzed using the one-way ANOVA. Categorical variables were described as number (%) and were compared by chi-square test.

Cox regression was used to estimate hazard ratios (HRs) and 95% confidence intervals (CIs) of incidence of CVD, heart disease and stroke associated with e-CRF as categorical variable by quartile, with the first quartile (Q1) as reference group, and as continuous variable. Models were separately constructed for males, females and all participants. Age, resident area, smoking status, drinking status, hypertension, TG, HDL-C, FPG and drugs used to treat for hypertension, dyslipidemia and diabetes were adjusted. The follow-up time for each participant was defined as the duration from baseline date to the diagnosis date of heart disease and stroke or the end of following up (Wave4, 2018), whichever came first. The incidence rate of CVD, heart disease and stroke was calculated by the number of incident events divided by the person-years. Proportional hazards assumptions were tested using standard Schoenfeld residuals and no violation was observed. The linear trend was tested by modelling e-CRF as an ordinal variable. Sensitivity analyses were carried out by excluding subjects whose outcomes occurred in the first year of follow-up to avoid reverse causation and using the equations of e-CRF developed by Nes et al. [9]. Subgroup analysis was performed by age group (≤ 60 and > 60 years).

A two-tailed *P* < 0.05 was defined as statistical significance. All statistical analyses were conducted using R software (4.2.0).

## Results

### Baseline characteristics of the participants

10,507 participants from CHARLS were included in this study. The mean age of the subjects was 57.2 years (SD 9.7) and 5,432 (51.6%) were females. The mean levels of e-CRF were 10.16 METs (SD 2.01) among all participants, and were significantly higher in males (11.68 METs) than in females (8.74 METs). After a median of 7 year’s follow-up, a total of 1,862 CVD, 1,409 heart disease and 612 stroke events were recorded.

All the participants were classified into four groups according to the quartiles of e-CRF by male and female, respectively. Table 1 presents the baseline characteristics of the participants by quartile of e-CRF overall and stratified by sex. Subjects in higher quartile were younger, less likely to be smokers, lower in BMI, WC, rHR, TG, SBP, DBP, FPG, and higher in HDL-C and PA (*P* < 0.001). Similar results were found after stratified by sex. There was no significant difference in drinking status across e-CRF quartile (*P* = 0.214).

**Table 2:** Associations between e-CRF and the risks of CVD, heart disease and stroke

e-CRF	Number of events	person year	Incident Density ^a^	HR (95%CI)	*P* value ^b^
**All population**					
**CVD**					
Q1	667	13,876	48.07	1(ref)	
Q2	494	14,917	33.12	0.81(0.71–0.93)	0.0032
Q3	395	15,357	25.72	0.71(0.61–0.83)	< 0.0001
Q4	306	15,624	19.59	0.61(0.51–0.73)	< 0.0001
*P*_trend_					< 0.0001
e-CRF (per 1-MET)				0.88(0.84–0.93)	< 0.0001
**Heart disease** ^c^					
Q1	487	14,216	34.26	1(ref)	
Q2	363	15,157	23.95	0.83(0.70–0.97)	0.0204
Q3	317	15,468	20.49	0.77(0.64–0.92)	0.0051
Q4	242	15,744	15.37	0.67(0.54–0.83)	< 0.0001
*P*_trend_					0.0002
e-CRF (per 1-MET)				0.89(0.85–0.94)	0.0001
**Stroke**					
Q1	256	15,132	16.92	1(ref)	
Q2	166	15,944	10.41	0.78(0.62–0.98)	0.0295
Q3	114	16,237	7.02	0.65(0.50–0.85)	0.0017
Q4	76	16,337	4.65	0.45(0.32–0.63)	< 0.0001
*P*_trend_					< 0.0001
e-CRF (per 1-MET)				0.88(0.82–0.95)	0.0017
**Men**					
**CVD**					
Q1	291	6692	43.49	1(ref)	
Q2	217	7173	30.25	0.84(0.69–1.03)	0.0973
Q3	174	7410	23.48	0.72(0.57–0.91)	0.0053
Q4	140	7581	18.47	0.66(0.51–0.87)	0.0030
*P*_trend_					0.0011
e-CRF (per 1-MET)				0.91(0.85–0.96)	0.0016
**Heart disease** ^c^					
Q1	195	6892	28.29	1(ref)	
Q2	142	7337	19.35	0.84(0.65–1.08)	0.1656
Q3	133	7489	17.76	0.84(0.64–1.12)	0.2354
Q4	98	7664	12.79	0.72(0.52–0.99)	0.0452
*P*_trend_					0.0619
e-CRF (per 1-MET)				0.93(0.86-1.00)	0.0547
**Stroke**					
Q1	128	7197	17.79	1(ref)	
Q2	92	7558	12.17	0.89(0.66–1.21)	0.4528
Q3	57	7777	7.33	0.59(0.41–0.87)	0.0068
Q4	49	7863	6.23	0.61(0.40–0.95)	0.0273
*P*_trend_					0.0049
e-CRF (per 1-MET)				0.90(0.82–0.99)	0.0318
**Women**					
**CVD**					
Q1	376	7184	52.34	1(ref)	
Q2	277	7744	35.77	0.79(0.66–0.96)	0.0148
Q3	221	7947	27.81	0.71(0.57–0.88)	0.0019
Q4	166	8043	20.64	0.58(0.45–0.75)	< 0.0001
*P*_trend_					< 0.0001
e-CRF (per 1-MET)				0.87(0.81–0.94)	0.0002
**Heart disease** ^c^					
Q1	292	7324	39.87	1(ref)	
Q2	221	7819	28.27	0.82(0.66–1.01)	0.0640
Q3	184	7979	23.06	0.72(0.57–0.92)	0.0092
Q4	144	8080	17.82	0.64(0.48–0.85)	0.0019
*P*_trend_					0.0015
e-CRF (per 1-MET)				0.87(0.80–0.94)	0.0008
**Stroke**					
Q1	128	7935	16.13	1(ref)	
Q2	74	8387	8.82	0.66(0.47–0.93)	0.0171
Q3	57	8460	6.74	0.71(0.47–1.05)	0.0852
Q4	27	8474	3.19	0.28(0.15–0.50)	< 0.0001
*P*_trend_					0.0001
e-CRF (per 1-MET)				0.86(0.75–0.98)	0.0270

^a^ Per 1000 person-years

^b^ Adjusted for age, resident area, smoking status, drinking status, hypertension, TG, HDL-C, FPG and drugs used to treat for hypertension, dyslipidemia and diabetes in male and female population and plus sex in all population

^c^ Heart disease was defined as heart attack, coronary heart disease (CHD), angina, congestive heart failure or other heart problems

Abbreviations: Q, quartile; CVD, cardiovascular disease; e-CRF, estimated cardiorespiratory fitness; MET, metabolic equivalent

**Table 3:** Sensitivity analyses on the association of the e-CRF with incident CVD, heart disease and stroke

	Q1	Q2	Q3	Q4	*P*_trend_	e-CRF (per 1-MET)
**excluding participants who developed events within 1 year**						
CVD	1(ref)	0.80(0.69–0.92)	0.71(0.60–0.83)	0.60(0.49–0.72)	< 0.0001	0.89(0.85–0.93)
Heart disease ^a^	1(ref)	0.81(0.69–0.96)	0.77(0.64–0.93)	0.66(0.53–0.82)	0.0002	0.90(0.85–0.95)
stroke	1(ref)	0.76(0.61–0.96)	0.62(0.47–0.82)	0.44(0.31–0.63)	< 0.0001	0.88(0.81–0.95)
**e-CRF developed by Nes** et al. ^**9**^						
CVD	1(ref)	0.82(0.71–0.93)	0.77(0.66–0.90)	0.65(0.55–0.77)	< 0.0001	0.91(0.88–0.94)
Heart disease ^a^	1(ref)	0.88(0.75–1.03)	0.83(0.69–0.99)	0.72(0.59–0.87)	0.0006	0.92(0.89–0.96)
stroke	1(ref)	0.75(0.59–0.94)	0.75(0.58–0.97)	0.54(0.40–0.72)	0.0001	0.89(0.84–0.95)
**< 60 years**						
CVD	1(ref)	0.90(0.74–1.08)	0.70(0.53–0.91)	0.50(0.36–0.70)	0.0001	0.85(0.79–0.90)
Heart disease ^a^	1(ref)	0.94(0.77–1.16)	0.82(0.61–1.11)	0.63(0.43–0.93)	0.0381	0.87(0.81–0.94)
stroke	1(ref)	0.75(0.52–1.09)	0.49(0.30–0.80)	0.29(0.16–0.51)	< 0.0001	0.79(0.71–0.88)
**≥ 60 years**						
CVD	1(ref)	0.97(0.79–1.19)	0.70(0.53–0.94)	0.64(0.45–0.92)	0.0142	0.91(0.85–0.97)
Heart disease ^a^	1(ref)	0.96(0.75–1.22)	0.70(0.49–0.98)	0.67(0.44–1.03)	0.0583	0.90(0.83–0.98)
stroke	1(ref)	0.87(0.61–1.24)	0.70(0.43–1.14)	0.71(0.40–1.25)	0.2270	0.95(0.85–1.07)

All models were adjusted for age, sex, resident area, smoking status, drinking status, hypertension, TG, HDL-C, FPG and drugs used to treat for hypertension, dyslipidemia and diabetes

^a^ Heart disease was defined as heart attack, coronary heart disease (CHD), angina, congestive heart failure or other heart problems

Abbreviations: Q, quartile; CVD, cardiovascular disease; e-CRF, estimated cardiorespiratory fitness; MET, metabolic equivalent

### Association between e-CRF and incidence of CVD, heart disease and stroke

Table 2 describes the incident densities and adjusted HRs of incidence of CVD, heart disease and stroke with e-CRF overall and stratified by sex. Overall, e-CRF was inversely associated with the risk of CVD (HR 0.88, 95%CI [0.84–0.93]) after adjusting for covariates. Compared with the first quartile (Q1) of e-CRF, significantly decreased risks of CVD were found with the HR of 0.81 (95% CI: 0.71–0.93) for Q2, 0.71 (95% CI: 0.61–0.83) for Q3, and 0.61 (95% CI: 0.51–0.73) for Q4. After stratified by heart disease and stroke, similar results were found as CVD. Similar results were found with the association between e-CRF and risk of heart disease (HR 0.83, 0.77, 0.67 for Q2, Q3, Q4; HR 0.88 per 1-unit MET increase; p < 0.05) and stroke (HR 0.78, 0.65, 0.45 for Q2, Q3. Q4; HR 0.88 per 1-unit MET increase; p < 0.05).

After stratified by sex, the associations were consistent among males and females. When using e-CRF was continuous variable, each one MET increment in e-CRF was associated with lower risk of CVD (males: HR 0.91, 95%CI [0.85–0.96], females: 0.87 [0.81–0.94]), heart disease (males: 0.93 [0.86-1.00], females: 0.87 [0.80–0.94]) and stroke (males: 0.90 [0.82–0.99], females: 0.86 [0.78–0.98]) in both males and females. The effect of association for heart disease and stroke were slightly weaker than that for CVD when using e-CRF as an ordinal variable, especially for males.

### Sensitivity analyses and subgroup analysis

The results of sensitivity analysis are presented in Table 3. The analysis that excluded the participants who developed events within one year after baseline and the analysis with e-CRF calculated by equations developed by Nes et al. both showed consistent results on the associations between e-CRF and incidence of CVD, heart disease and stroke [9]. Subgroup analysis that stratified by age (< 60 and ≥ 60 years) found significant associations for CVD and heart disease in both age groups, however the effect of association for stroke in subjects aged ≥ 60 years were slightly weaker.

## Discussion

In this study, we explored the association between e-CRF and risk of CVD, heart disease and stroke with data from CHARLS, a representative cohort study from China. We found that higher level of e-CRF was associated with reduced risk of CVD, heart disease and stroke for both males and females. The associations between e-CRF and incidence of CVD, heart disease and stroke were robust after excluding participants who developed events within a year and stratifying participants into the mid-age (45–60 years) and the eldly (> 60 years). Furthermore, the associations based on CRF estimated by Nes et al. were consistent with that based on Jackson et al. [9, 14].

The reduction in CVD risk associated with increased e-CRF in our study are comparable to those studies that explored the association between measured CRF and CVD. In this study, each one MET increment in e-CRF was associated with a 9% (95%CI: 4-15%) and 13% (6-19%) lower risk of CVD, a 7% (0-14%) and 13% (6-20%) lower risk of heart disease and a 10% (1-18%) and 14% (2-22%) lower risk of stroke in males and females, respectively. In a meta-analysis including 84,323 participants based on 33 eligible studies, the risk of CHD/CVD decreased 15% (12-18%) as per 1-MET higher level of CRF [24]. Furthermore, the association remained robust after sex-specific and age-specific subgroup analyses, which is consistent with this study. Recently, a meta-analysis involving 392,240 individuals based on 18 prospective cohort studies showed the risk of CVD mortality was decreased by 13% (9-17%) with each one-MET increase in CRF and the dose-response associations were consistent in subgroup analyses across sex [4].

Studies looking at the associations between e-CRF and incidence of CVD are limited. Several studies have showed inconsistent associations between males and females. Compared to males with low e-CRF, males with high level of e-CRF estimated from longitudinal models were 29% less likely to develop CHD during a 12 years follow-up from ACLS prospective cohort, however females were excluded because of limited incident cases [19]. In another study based on ACLS cohort, both medium and high CRF were inversely with risks of non-fatal CVD events and CVD mortality in males. In females, only high e-CRF was significantly associated with non-fatal CVD risk [15]. In Nord-Trøndelage Health study (HUNT), e-CRF was associated with decreased risk of first acute myocardial infarction events among females but not in males [20]. In another HUNT study focusing on the association between e-CRF and risk of atrial fibrillation, the highest risk reduction of AF was 31% and 47% in the fourth quintile of eCRF when compared with the first quintile in males and females, respectively [25]. In our study, only the effect of association for heart disease and stroke were slightly weaker than that for CVD when using e-CRF as an ordinal variable, especially for males. Sex-specific algorithms or differences in included covariates and ethnicities may result in these inconsistent findings. Our study provided relative stable evidence for applying the longitudinal equations to estimate CRF and further exploring the associations between e-CRF and incidence of CVD events. All the variables in the estimating equations could be easily available in hospitals and large-population investigation. Considering the multiple limitations of measuring CRF, e-CRF obtained from non-exercise longitudinal algorithms can be a cost-effective way of assessing CRF as a useful and simple tool to identify subjects who are in high risks of CVD in routine clinical practice.

This study has several strengths. First, the data are from a nationally representative cohort with large sample size, rigorous research design and high-quality data. Second, compared with other models based on cross-sectional studies, the application of longitudinal equations may provide a more accurate estimation of e-CRF. Several limitations in this study should also be noted. First, the information of outcome was from self-reported questionnaire. Second, the effects of e-CRF for each kind of heart disease may be underestimated due to using overall heart disease as one outcome. Third, physical activity was from self-report, which may induce recall bias into the estimation of e-CRF. Lastly, other possible variables (muscle strength, maximal voluntary ventilation and maximal cardiac output) that may affect e-CRF aside from the components used in the longitudinal models were not examined in our study.

## Conclusions

Non-exercise estimated CRF based on the commonly collected health indicators was inversely associated with the incidence of CVD, heart disease and stroke in both males and females in a representative sample of the Chinese population. The estimation method could be used to predict the risk of CVD events in clinical practice or large-population studies. Further research is needed to explore the associations between e-CRF changes and CVD events.

## Data Availability

The authors thank the China Center for Economic Research, National School of Development, Peking University for providing the data. The data of CHARLS could be download from the website of CHARLS CHARLS (pku.edu.cn).

## Abbreviations

Q: Quartile
SD: Standard deviation
BMI: Body mass index
WC: Waist circumference
rHR: Resting heart rate
SBP: Systolic blood pressure
DBP: Diastolic blood pressure
TG: Triglycerides
HDL-C: High-density lipoprotein cholesterol
FPG: Fasting plasma glucose
e-CRF: Estimated cardiorespiratory fitness
MET: Metabolic equivalent
